# Current concepts in parathyroid carcinoma: a single Centre experience

**DOI:** 10.1186/s12902-019-0368-1

**Published:** 2019-05-29

**Authors:** Valentina Ferraro, Lucia Ilaria Sgaramella, Giovanna Di Meo, Francesco Paolo Prete, Francesco Logoluso, Francesco Minerva, Marica Noviello, Giuseppina Renzulli, Angela Gurrado, Mario Testini

**Affiliations:** 1 Department of Biomedical Sciences and Human Oncology – Unit of Endocrine, Digestive and Emergency Surgery, University Medical School of Bari, Bari, Italy; 2 Department of Emergency and Organ Transplant, University Medical School of Bari, Bari, Italy; 3 Interdisciplinary Department of Medicine, University Medical School of Bari, Bari, Italy

**Keywords:** Parathyroid Carcinoma, Parathyroidectomy, Endocrine surgery, Hyperparparathyroidism, Hypercalcemia, MEN 1, Diagnosis

## Abstract

**Background:**

Parathyroid carcinoma is a rare neoplasm that may present sporadically or in the context of a genetic syndrome. Diagnosis and management are challenging due to the lack of clinical and pathological features that may reliably distinguish malignant from benign disease.

**Methods:**

From January 2013 to December 2017, from 358 consecutive patients affected by parathyroid diseases, 3 patients with parathyroid carcinoma were treated at our academic Department of General Surgery. We present our experience as illustrative of the different features of clinical presentation of parathyroid carcinoma and review its management considering the recent relevant literature.

**Results:**

*Case 1:* A 62-year-old man was hospitalized for left-sided palpable neck mass, hypercalcemia and elevated PTH. US-guided FNA was suspect for parathyroid carcinoma. A large cystic mass was excised in bloc with total thyroidectomy and central neck dissection. Genetic studies framed a pathologically confirmed parathyroid carcinoma within MEN1 syndrome. *Case 2:* A 48-year-old woman with hypothyroidism had total thyroidectomy performed for a suspect for right follicular thyroid lesion. Pathology revealed parathyroid carcinoma. *Case 3:* A 47 year-old man was admitted for hypercalcaemic crisis and renal failure in the context of PHPT. A lesion suggestive on US and MIBI scan for parathyroid adenoma in the right lower position was removed by mini-invasive approach. Pathology revealed parathyroid cancer and patient had completion hemythyroidectomy and central neck dissection.

**Conclusion:**

Parathyroid cancer is a particularly rare endocrine malignancy, however it should be suspected in patients with primary hyperparathyroidism when severe hypercalcemia is associated to cervical mass, renal and skeletal disease. Parathyroid surgery remains the mainstay of treatment. Radical tumour resection and expedited treatment in a dedicated endocrine Center represent crucial prognostic factors.

## Background

Parathyroid carcinoma (PC) is an uncommon malignancy, less than 1000 recorded cases reported in literature since described by de Quervain in 1904 [1] as a non-functioning metastatic carcinoma [2–6]. With an estimated prevalence of 0.005% of all cancers [7, 8] PC is the rarest endocrine cancer, and accounts for 0.5–5% of all circumstances of primary hyperparathyroidism (PHPT) [6, 7, 9–13]. Pathogenesis of PC is unknown. It may be sporadic or occur in the context of a genetic endocrine syndrome as in hyperparathyroidism/jaw tumour syndrome (HPT-JT), Multiple Endocrine Neoplasia type 1 (MEN1), type 2A (MEN2A), and familial isolated hyperparathyroidism (FIHP) [14–16]. PC poses a diagnostic challenge because of the absence of characteristics that allow definite distinction of malignant from benign disease. Due to its rarity, there is no general consensus on treatment and follow-up. In a previous study we addressed parathyroid carcinoma arising in the context of MEN1syndrome [17]. The aim of this study is to appraise the recent literature on management of PC with respect to three illustrative cases of PCs treated consecutively at our Institution over the last 5 years.

## Methods

From January 2013 to December 2017, from 358 patients affected by parathyroid (PTs) disease, 3 patients (0.8%) were managed for PC (1 Female and 2 Male; mean age: 52.33, range: 47–62) at our Academic Department of General Surgery. We retrospectively reviewed clinical presentation of PC, along with diagnosis, management and follow up.

## Results

Case 1: a 62-year-old gentleman with a background of nephrolithiasis, constipation, thalassaemia minor and non-insulin-dependent diabetes was admitted with an asymptomatic, left-sided large neck mass, raised serum calcium (2,92 mmol/L), elevated PTH (391.7 pg/ml) and polyuria. Doppler ultrasound scan showed a large cystic mass contiguous to the left lobe of a multinodular thyroid and to the oesophagus, partially retrosternal. CT confirmed a 9-cm lesion extending into the upper mediastinum. FNAC demonstrated a PT neoplasm. The mass was excised in bloc with total thyroidectomy and central neck dissection (CND). Microsurgical technique (loupe magnification 3*×*) was used to dissect the mass off the strap muscles, left recurrent laryngeal nerve (RLN) and oesophagus, as the tumour was adherent by proximity to these structures. Pathology confirmed PC with no thyroid or lymph node involvement.

A hereditary disease was suspected when the patient’s brother was diagnosed with PC in the interim, and a germline mutation (c.1252 G > A) was found in the MEN1 gene of the patient and of two relatives. The patient was assessed for lesions related to MEN 1. Magnetic Resonance Imaging (MRI) of the head did not document lesions in the pituitary. Pancreatic lesions suggesting MEN1-related neuroendocrine tumours were identified on endoscopic US, while CT scan detected a non-functional adrenal nodule. At 5-year follow-up, the patient shows normal calcemia and is free from recurrence. Features of this case were presented in a previous publication [17].

Case 2: a 48-year-old woman with a background history of essential hypertension was referred to our Department for thyroid disease. Clinical examination was otherwise normal. TSH (thyroid-stimulating hormone) was 5.67 mUI/L (range 0.36–3.74 mUI/L), while FT4 (free triiodothyronine T4), FT3 (free triiodothyronine T3), serum calcium, phosphorous, calcitonin, and thyroglobulin levels were normal. PTH was not investigated. Neck US scan showed a 33x15x17 mm solid nodule with fluid areas located at the posterior margin of the right lobe of a normally sized thyroid; no adenopathy was found. FNA supported a working diagnosis of follicular thyroid lesion, and total thyroidectomy was scheduled. During surgery a solid grayish mass of 2.2 × 1.6 × 1 cm was found behind the middle third of the right lobe of the thyroid, in close contact with trachea and the oesophageal wall, and was carefully dissected off by microsurgical technique. No distinct superior PT gland could be found. The patient was uneventfully discharged 2 days after surgery. Pathology revealed intrathyroidal PC of the right thyroid lobe with a cystic component, fibrosis, endocapillary proliferation but no invasion of the thyroid gland. The 30-month follow-up did not show residual-recurrent disease.

Case 3: a 47 year-old man, admitted as a medical emergency for renal failure, hypercalcaemic crisis (5.33 mmol/L, n.v. = 2.25–2.67 mmol/L) and symptomatic nephrolithiasis, was referred to our Department for PHPT. Physical examination was unremarkable. Aside severe renal failure and hypercalcemia, laboratory tests showed hypophosphataemia (0.55 mg/dL, n.v. = 2.5–4.50 mg/dL) and high PTH (919.0 p/mL; n.v.: 6.4–52 pg/mL). Excreted 24-h urinary calcium was 494 mg/24 h (n.v. = 42–353 mg/24 h), phosphate excretion was normal and there was proteinuria. US scan identified a hypoechoic 17 × 16 mm nodule on the posterior margin of the inferior right lobe suggestive for parathyroid adenoma (PA). Technetium-99 m-sestamibi (MIBI) dual-phase scan documented increased uptake at the level of the right inferior PT gland. The patient underwent right inferior minimally invasive video-assisted parathyroidectomy. During surgery a 4 × 2.5 × 2 cm solid brown mass was found, and after resection intraoperative PTH dropped by more than 50%. Postoperative course was uneventful, with decreasing PTH levels, and the patient was discharged on the second postoperative day. Pathology revealed a PC with microscopic aspects of vascular invasion and surrounding adipose tissue infiltration. The patient was scheduled for completion surgery while showing increasing PTH levels (429.0 pg/mL n.v.: 6.4–52 pg/mL). Hemythyroidectomy and CND were performed, with uneventful recovery and discharge on postoperative day 3. Final pathology showed no thyroid or lymph nodes involvement.

## Discussion

### Epidemiology

PCs are extremely rare tumours, in fact one of the rarest of all human cancers with an incidence of 0.015 per 100,000 population and a prevalence of 0.005% in the United States [8, 18]. HPT has been estimated in 0.017 to 5.2% of the cases of PC, accounting for < 1% of patients with PHPT in many series [18–21]. There is no gender or race dominance, and usually PC occurs a decade earlier than PA, at a mean age of 45-51 years [8, 18]. The 90% of PC are hormonally functional, with most patients presenting with hypercalcemia as the initial manifestation of the disease [5]; to date, 38 cases of non-functional PC are have been reported in literature, mostly presenting during the sixth or seventh decade [22].

### Etiology

Etiology of PC is unknown, and there is no evidence of PC arising from transformation of pre-existing PT lesions [23]. Instead, aberrant microRNA expression profile and methylation signature recently identified in PCs, provide evidence that malignancies of the parathyroid gland are distinct entities from benign lesions [24]. PC can be sporadic or be part of a genetic syndrome [25], Sporadic PC has been linked with exposure to external radiation [2, 26, 27] and rarely with secondary and tertiary HPT due to chronic renal failure [28–30]. An association of PC, synchronous or metachronous to a history of PT glands hyperplasia, PA or thyroid cancer with concomitant PA has also been reported [21, 31, 32]. PC in the context of a genetic syndrome may occur in 15% of individuals with HPT-JT [16, 33, 34], in 1% of FIHP patients [8, 35, 36], has rarely been reported in MEN2A syndrome [37] and represents 0.28–1% of all surgically treated HPT in MEN 1 [38, 39]. The most frequent genetic anomalies associated with PC are inactivating somatic mutations of parafibromin gene (CDC73/HRPT2); other abnormalities include altered expression of the p53 and retinoblastoma proteins, and oncosuppressor genes on chromosome 13 [40–42]. In the run-up to a full characterization of PC at molecular level, up to one fifth of cases from a recent large single-institution series showed alterations of the PI3K/AKT/mTOR oncogenic pathway, and about a third had Cyclin D1 overexpression supported by its relative CCND1 gene amplification [43].

### Clinical features

PC typically displays an indolent, albeit tenacious, course; patients often exhibit symptoms and complications of severe PHPT such as anxiety, depression, weakness, weight loss, bone and renal disease, abdominal pain, nausea, pancreatitis and peptic ulcer [4, 6, 8, 44].

At presentation 50% of patients show both manifestations of renal and skeletal involvement including osteopenia, osteoporosis, osteofibrosis, osteitis fibrosa cystica, subperiostal resorption, “salt and pepper” skull and pathologic fractures (up to 90% of symptomatic patients) [6, 14, 18, 21, 44]. Renal disease manifests mostly as nephrolithiasis and renal insufficiency with a reported prevalence of 56 and 84%, respectively [45]. A highly suggestive clinical sign is hoarseness due to RLN palsy [46]. Some patients, as Case 3, may present with hypercalcaemic crisis.

Non-functioning PCs (less than 10%) usually present at a more advanced stage with symptoms of compression/invasion of adjacent structures [22, 31, 47–49]: neck mass and/or dysphagia (up to 80% of non-functioning cases), hoarseness or dyspnoea [7, 22, 50]. Physical examination may be unrevealing, as in Case 2, although a palpable cervical mass has been described in 30 to 76% of patients [21, 45, 51, 52]. Entirely asymptomatic PC has been reported in 7 to 46% of patients [7, 45, 53, 54].

### Laboratory tests

Serum calcium levels in PC have been noted to be frequently higher than 3.5 mmol/L (vs. < 2.8 mmol/L in benign disease), while PTH serum levels have commonly been 3 to 10 times as high as the upper normal limit (benign PT disease may show more modest increases) [7, 14, 26, 45, 50, 55], although there is no agreed threshold of PTH and serum calcium level to define PT malignancy. Alkaline phosphatase has been shown to be significantly higher in PC with respect to benign disease, and levels < 300 IU/L are unlikely to be associated with PC [54]. Serum and urinary human chorionic gonadotropin levels (along with its its hyperglycosilated isoform) may be abnormally elevated in PC, but not in PA [56].

### Imaging

As in benign disease, imaging is useful for tumour localization, but cannot reliably discriminate benign from malignant disease. The use of more than one imaging method, most commonly a combination of MIBI and neck US scan, increases diagnostic sensitivity and accuracy [5, 14, 57, 58]. Some US features may anticipate malignancy and help identify enlargement of lymph node or invasion into nearby structures [59, 60]: size > 3 cm should raise the suspicion of PC [61], as in Case 1; PCs are more likely to be inhomogeneous, hypoechoic, with lobulated/non-circumscribed margins, degenerative changes, intra-nodular calcification, suspicious vascularity, a thick capsule and local invasion; on the contrary, PAs appear as homogenous, smooth, and smaller masses [61–64]. MIBI allows localization and hence distinction between eutopic and ectopic PT tissue, as well as identification of recurrent disease [46, 58, 65]. Both CT and MRI may provide information about lesion extension, eventual invasion into surrounding structures, lymph nodes or distant metastasis [5, 60]; CT usually shows low sensitivity in detecting PC [66], while MRI is adequate for soft tissue studies [67]. Few studies are available on the use of 18F-fluorodeoxyglucose positron emission tomography (FDG PET), aimed at the early identification of metastases/recurrence [67–70].

### Cytology, histopathology

Cytological and histopathological diagnosis are challenging [17, 20]. If PC is pre-operatively suspected, FNA should be avoided due to the risk of tumour dissemination along the needle tract, with higher risk of recurrence; biopsy has resulted in capsular rupture and PT tissue diffusion [5, 66, 71, 72]. In 1973, *Schantz* and *Castleman* first reported morphologic principles defining PC: fibrous bands arranged in a trabecular design, capsular invasion [73–75], vascular invasion, and mitotic activity [23]. Capsular and vascular invasion have been linked with tumour recurrences and distant metastases, and are considered the sole pathognomonic markers of malignancy [76, 77]. Poorer prognosis is also associated to aneuploid tumour DNA pattern [26, 57, 78].

The histological features of non-functioning and functioning PC are similar. These tumours, mainly containing clear or oxyphil cells, are large and can be misdiagnosed as cancers of the thyroid or of the thymus. Immunohistochemistry may improve diagnostic accuracy of PC as also suggested by the American Association of Endocrine Surgeons Guidelines for Definitive Management of Primary HPT. Non-functional PC may stain positively for intracellular PTH and chromogranin A, while not displaying calcitonin, thyroglobulin and thyroid transcription factor 1 [47]. Ki67 greater than 5% suggests PC. In general, a panel of immunohistochemistry is more accurate than use of any single marker per se to diagnose PC. A number of oncogenes and onco-suppressor genes have been related to PC [79]. Somatic mutation of CDC73, a tumour suppressor gene associated with parafibromin expression, is carried by up to 70% of PC [80]; such mutation is rarely found in benign sporadic PAs (0.8%) [29, 81–83]. Germline mutations are presents in a third of cases of PC [84], suggesting HPT-JT syndrome in a subgroup of patients (germline mutations of CDC73 gene have been responsible for HPT-JT and occasionally FIHP) [29, 33, 49, 81, 82, 85–92]. Presence of CDC73 gene mutation with negative parafibromin staining, very rarely found in Pas, increases the probability of malignancy [93–102].

Other genes or their protein products, as Rb, p53, BRCA2 (breast cancer susceptibility) and PRAD1 (cyclin D1/PA 1 oncogene) [47, 103–105] have been linked with PC, although none of them could consistently and reliably distinguish between benign and malignant disease [105]. Anomalous expression of microRNAs has also been described in PC [106].

### Intra-operative diagnosis

Intra-operative findings have been described that raise the suspicion of PC. In most series, the median maximal diameter of PC is 3–3.5 cm (< 10% larger than 4 cm) -as in Case 1-, compared with approximately 1.5 cm for PA [8, 12, 18, 107]. PC colour may range from greyish to white. It has been reported that 21% of PC displays a cystic component (Case 1) [5, 31]. These tumours are firm, often showing a stony-hard consistency, occasionally lobulated and usually surrounded by a dense fibrous capsule. They invade or adhere to adjacent structures, such as thyroid, strap muscles, the ipsilateral RLN, oesophagus and trachea (Case 1) [10, 57, 107, 108]. PAs are instead usually smaller and soft, of a reddish-brown colour, round or oval in shape [21, 107].

### Stage information

No consensus clinical and pathological staging system for PC has been universally adopted, as there is no correlation of tumour diameter or lymph node status with survival, and the disease is rarely diagnosed preoperatively or even intraoperatively [8, 11, 27, 109, 110]. Schulte et al have proposed a staging system based on two classification schemes, the differentiated classification as in the framework of TNM classification, and high-risk/low-risk classification that seems to show a significant power of prediction for survival and recurrence [111, 112].

### Surgical treatment

Historically almost 96% of patients with PC have been surgically treated [8, 12], and surgery is the only effective therapy to control hypercalcaemia and reduce tumor load, both at initial resection and at the time of recurrence of metastasis [18, 21, 113]. Optimal management of calcaemia is important prior to surgery [114]. At the time of the first operation, complete resection with microscopically negative margins and intact tumour capsule is the treatment of choice to achieve the best chance of cure. Reported surgical interventions fall into two main categories: *in-bloc* and local only excision. Local excision includes pericapsular excision only of the affected PT [112]. However most authors concur that an *en bloc* excision at the initial operation reduces the need for repeat surgery and improve survival outcomes; en bloc excision consists of resection of the tumour, ipsilateral thyroid lobectomy and resection of adjacent cervical muscles, paratracheal lymphatic and alveolar tissue, and -if involved- an RLN segment [13, 79, 110]. En bloc resection carries a moderate risk when performed by dedicated endocrine oncologic surgeons [112]. Overall, 8% local recurrence rate after *en bloc* resection compares with 51% after standard parathyroidectomy [27]. However, only 12.5% of patients have been reported to have undergone *en bloc* resection compared with 78.5% receiving parathyroidectomy alone, most likely because of their PC not being identified either preoperatively or even intraoperatively [12]. This situation is illustrated by the second case that we presented, where there were no preoperative or intraoperative signs raising the suspicion of PC, so surgical strategy was that for a benign disease in first instance, and only upon definitive pathological diagnosis completion surgery was planned. Neck re-exploration, along with removal of the tissue surrounding the removed PC is recommended when diagnosis is made after the initial procedure [21].

In case of thyroid surgery that encompasses parathyroid resection for PT incidentaloma (Case 3), *en bloc* resection of PC diagnosed in retrospect is possible as long as there is removal of all structures immediately adjacent to the affected PT at the time of first surgical approach. Goal of surgery is in fact complete resection with clear gross margin ad an intact capsule.

There is no clear evidence that performing prophylactic CND or lateral neck dissection, may improve survival [8, 12, 14, 31, 115, 116]. On the other hand CND has been proposed at the first surgical approach in all patients with PC, on the basis that central compartment has been involved in up to 10% of cases, and that omitting a central and/or ipsilateral jugular compartment dissection might carry a 1.5 to 2 times higher risk of both 5-year recurrence and death [111]. Lymph node excision -beyond that necessary for in bloc excision of the primary tumour- is not currently indicated unless nodal disease is clinically documented by enlarged or firm nodes, particularly in the VI, III and IV levels [18].

After PC resection intra-operative PTH levels can fall significantly, although often not as quickly as with benign disease; no PTH may indicate incomplete tumour resection, or concomitant multiple gland hyperplasia [117], or even multiple PCs [118]. Exploration of all four parathyroids may be required as both carcinoma in multiple glands and the concurrent presence of PC and PA has been documented in the literature [119, 120]. Use of intraoperative PTH assay is subject to surgery scheduled for PT disease, and in case of PT incidentaloma the necessary kit for PTH reading may not be available.

### Follow-up

The indication that all hyperfunctioning tissue has been removed after surgery is restoration of normal calcemia. Nevertheless, follow-up monitoring should be life-long because risk of multiple relapses may be relatively high over prolonged intervals of time after surgery [53]. During follow-up patients with functioning PC should undergo serum calcium, PTH levels and regular US surveillance, whereas those with non-functional PC should only rely on imaging studies.

Post-operative diagnosis of PC in a patient formerly suspected to have benign pathology, as in Cases 2 and 3, is a common clinical situation. If the macroscopic features of the tumour are typical of a PC and histology shows extensive vascular or capsular invasion, reintervention with resection of the adjacent structures should be considered: vascular invasion has been reported as an important independent predictor associated with recurrence and haematogenous spread [111, 112]. Repeat surgery should also be considered in patients with persistent or worsening hypercalcemia, after appropriate localization studies [21, 121]. Instead in the presence of normocalcemia and an equivocal histology there is no indication to immediate reoperation, as complete resection of the tumour may have been curative.

### Metastasis and local recurrence

Lymph node and distant metastases at the time of diagnosis are uncommon, occurring respectively in 1–6% and 2–4% [9, 11, 12, 26, 27, 45, 50, 53, 122]. About one half of the patients has experienced relapse 2 to 5 years after the initial resection, usually presenting with a slowly increasing serum calcium and PTH level in functioning PCs [22, 47, 110, 123]. Distant metastases occur in about 25% of patients [26, 124] during follow-up, although incidence of relapse has been higher in recent series [18]. The disease-free interval to the appearance of metastatic disease may be up to 20 years [94, 110, 124], with most common sites in the lungs (40%), liver (10%) and, very rarely, bone, pleura, pericardium, and pancreas [20, 21].

The clinical course is usually indolent, and some patients survive for years even after diagnosis of metastatic disease. Disease progression is usually marked mainly by the clinical manifestations of hypercalcemia and its related complications [21]. In some cases the first indicator of tumour malignancy may be recurrent or metastatic disease in a previous parathyroidectomy for hypercalcemia [53].

Recurrence is typically regional, accounts for approximately two thirds of relapses [26] and is often difficult to detect as it may be small, multifocal, or grow in the scar from the previous surgery. Approximately one half of the patients with recurrence also present distant metastases [124].

The primary treatment for tumour relapse is the surgical removal of the local recurrences and distant metastases whenever possible, even though repeat resection [8, 18, 45, 125–128]. In retrospective series aggressive surgical resection of recurrent disease has been associated with up to 30% increase in long-term survival [124, 126]. Resection of tumour deposits in the neck, lymph nodes, liver or lungs, even when very small, may provide significant palliation, rendering hypercalcemia more medically manageable, and resulting in normocalcemia that may range from months to years [8, 9, 26, 45, 94, 125, 127, 129].

At least two independent non-invasive preoperative localization studies should be performed in all patients prior to reintervention: US is effective in localizing local metastases, especially those involving neck lymph nodes, while MIBI scan can detect both local and distant metastases, which may also be visible on CT and MRI [65, 130, 131]. If previous imaging studies fail to locate the sites of recurrence or show equivocal results, selective venous catheterization with PTH sampling could be considered [57, 104]. FNA of equivocal lesions should be avoided because of the potential for dissemination [72].

In redo neck surgeries the cumulative risks for surgical complication greatly increase (up to 60%) and the RLN injury rate is higher (up to 38%) [31, 104, 132].

In patients affected by genetic syndromes as HPT-JT and MEN1, recurrent hypercalcemia following initial surgery may be a sign of recurrence of the primary tumour and/or distant metastases, metastases or disease in a further PT [133].

Radiofrequency ablation, either alone or in combination with arterial embolization has been effective in treating liver and pulmonary metastases, and has shown ability to improve serum PTH and calcium levels [134, 135].

Percutaneous US-guided alcohol injection has been reported in local recurrences, producing improvements in calcium and PTH levels in the short term [136]. However, in view of complications associated with ethanol toxicity and RLN injuries [137] its use in the central neck region should be carefully considered.

### Chemotherapy and radiotherapy

Non-operative therapies for PC generally have poor results [18, 21, 53, 113]. Chemotherapy is usually ineffective. There are anecdotal reports of short-term remissions with dacarbazine as a single agent [110], while therapy with combined cyclophosphamide, flurouracil and dacarbazine, or a combination of methotrexate, doxorubicin, cyclophosphamide, and lomustine in patients with metastatic PC has shown no overall survival gain [138–140].

The results of radiotherapy are also usually non satisfactory [45] and currently no evidence supports radiation therapy as a primary modality of treatment. Despite these neoplasms show radiation resistance, some investigators have supported use of radiation to improve on local recurrence rates and overall survival in small series [8, 45, 51, 53, 79, 141]*.* Reported experience on palliative radiotherapy is even scarcer.

### Novel therapies

Single case reports have provided some evidence that anti-PTH immunotherapy may help prolonged control of hypercalcaemia unresponsive to conventional treatments, and control tumor growth, in some case achieving reduction of metastatic tumor burden [142–144]. Use of biologic agents aimed at blocking Cyclin D1 pathway, and development of telomerase inhibitors, so far tested in vitro, may represent therapeutic options in future [145].

With regards to the role of immune microenvironment in parathyroid neoplasms there is paucity of material in the recent literature, and potential for use of immunotherapy in PC remains unexplored. Some authors have suggested possible benefit from immunotherapy basing on the expression in the microenvironments of PCs of lymphocytes infiltrating the tumor, death ligand 1 and CD68+ as potential biomarkers target in cancer therapies [106].

### Prognosis

The prognosis of PC is variable. Overall survival rates are generally better than in most solid tumours, with 76 to 85% and 49 to 77% patients alive respectively at 5-year and 10-year follow-up [8, 12, 31, 53, 110].

The lack of a universally approved staging system for PC does not allow to offer a reliable prognostication system to patients. Observations on the prognosis of PC mainly derive from longitudinal single-institution studies and small series [5, 145]. Schulte et al show that the relative risk of cancer-related death is substantially higher in high-risk patients -in fact almost exclusive to such patients, given their 12.8 times higher relative risk of tumour relapse- [112]. Complete cure is unlikely with tumour recurrence, although palliative surgery can still afford prolonged survival.

The best prognosis is tied to complete tumour resection at the initial surgery, with survival rates up to 90% at 5 years and 67% at 10 years [146]. In studies where complete pathology reports on lymph node status were available, nodal status was shown to be a prognostic factor associated with mortality [31, 122].

Independent negative factors for survival have also been age, sex, distant metastases, time interval to first recurrence, higher calcaemia at recurrence, use of multiple calcimimetic agents, amount of recurrences in the neck, simple parathyroidectomy as initial surgery, inability to achieve complete tumour resection, and tumour DNA aneuploidy [12, 31, 53, 110]. In addition, patients with a tumour harbouring a CDC73 mutation, or loss of CASR or parafibromin expression presented worse survival outcomes [101, 147]. The prognosis of non-functional PC also appears to be worse since they are often diagnosed in advanced stages, already showing local invasion, nodal and/or distant metastasis. Cancer-related death in non-functional PC patients is primarily due to the burden of regional disease and to distant metastases, while in functional cancers it is usually due to renal failure, cardiac arrhythmias or pancreatitis resulting from uncontrollable hypercalcemia [31, 148].

## Conclusion

Despite its rarity, in the presence of HPT showing severe hypercalcemia, large cervical mass and concomitant renal and skeletal disease, PC should be suspected. Complete surgical resection at the earliest possible time is the optimal treatment, and patients with a suspicion of PC should be considered for referral to a Centre dedicated to PT surgery, to improve outcomes and offer the best chance of cure. Further multi-centric studies are needed to standardize surgical management of PC with the aim to improve results.

## Abbreviations

CND: Central neck dissection
CT: Computed Tomography
FIHP: Familial isolated hyperparathyroidism
FNA: Fine Needle Aspiration
FNAC: Fine Needle Aspiration Cytology
FT3: Free triiodothyronine T3
FT4: Free triiodothyronine T4
HPT: Hyperparathyroidism
HPT-JT: Hyperparathyroidism jaw tumour syndrome
MEN 2A: Multiple Endocrine Neoplasia type 2A
MEN1: Multiple Endocrine Neoplasia type 1
MIBI: Technetium- 99 m Sestamibi scintigraphy
MRI: Magnetic Resonance Imaging
PA: Parathyroid Adenoma
PC: Parathyroid carcinoma
PHPT: Primary hyperparathyroidism
PT: Parathyroid
PTH: Parathyroid Hormone
RLN: Recurrent laryngeal nerve
TSH: Thyroid-stimulating hormone
US: Ultrasound
